# Temporal lung changes on thin-section CT in patients with COVID-19 pneumonia

**DOI:** 10.1038/s41598-020-76776-x

**Published:** 2020-11-12

**Authors:** Zhiyan Zhang, Runhui Tang, Heyang Sun, Haiyang Dai, Kangyin Chen, Xinmiao Ye, Wei Ye, Shengkai Li, Bowen Lan, Li Li, Chun-Quan Ou

**Affiliations:** 1 Department of Medical Imaging, The Central People’s Hospital of Huizhou, No. 41, Eling Road North, Huicheng District, Huizhou, 516001 Guangdong Province China; 2 Department of Urology, The Central People’s Hospital of Huizhou, No. 41, Eling Road North, Huicheng District, Huizhou, Guangdong Province China; 3 State Key Laboratory of Organ Failure Research, Department of Biostatistics, Guangdong Provincial Key Laboratory of Tropical Disease Research, School of Public Health, Southern Medical University, No. 1023, Shatai Road South, Baiyun District, Guangzhou, 510515 Guangdong Province China

**Keywords:** Infectious diseases, Viral infection

## Abstract

We examined characteristics of chest CT across different time periods for patients with COVID-19 pneumonia in Huizhou, China. This study included 56 COVID-19 patients with abnormal CT acquired between January 22 and March 3, 2020. The 141 scans of 56 patients were classified into four groups (Groups 1–4) based on dates on which scans were obtained at the 1st, 2nd, 3rd week or longer than three weeks after illness onset. Forty-five patients with follow-up scans were categorized into four groups (Groups A–D) according to extent that lesions reduced (≥ 75%, 50–75%, 25–50% and < 25%). Ground-glass opacities (GGO) was prevalent in Groups 1–4 (58.1–82.6%), while percentages of consolidation ranged between 9.7% in Group 4 and 26.2% in Group 2. The highest frequency of fibrous stripes occurred in Group 3 (46.7%). Total CT scores were on average higher in Groups 2–3. Among 45 follow-up patients, 11 (24.4%) of them recovered with lesions reducing ≥ 75%, with the lowest median age and total CT scores on admission. There are temporal patterns of lung abnormalities in COVID-19 patients, with higher extent of lesion involvement occurring in the 2nd and 3rd week. Persisting lung changes indicate some patients may need isolation after discharge from hospital.

## Introduction

Large outbreaks of coronavirus disease 2019 (COVID-19) occurred globally. As of 10 AM CEST, 27 June 2020, there have been 9,653,048 confirmed cases of COVID-19 and in total of 491,128 deaths throughout the world, which far exceeded the burden of severe acute respiratory syndrome (SARS) and Middle East respiratory syndrome (MERS)^1,2^. COVID-19 pneumonia is caused by infections of severe acute respiratory syndrome coronavirus 2 (SARS-CoV-2), which is the seventh member of coronaviruses. A certain number of patients with COVID-19 initially presented with fever, cough and myalgia or fatigue, and about half of the patients developed dyspnea^3^. As the knowledge of COVID-19 pneumonia has been accumulated, professional consensus, guidelines, and criteria have been established steadily to facilitate the diagnosis and treatment of COVID-19 pneumonia^4^.

For screening COVID-19 patients, the information on epidemiology history, clinical symptoms, laboratory findings and chest imaging were always recorded. At present, the gold standard for diagnosis of COVID-19 is the result of real-time polymerase chain reaction (PCR), which is performed on specimens taken from throat swabs, sputum, lower respiratory tract or blood^5^. However, the nucleic acid test is time consuming and has a high false negative rate at the early stage of infections^6^. Lack of cellular material and improper extraction of nucleic acid partially results in the high false negative rate^7^. As a supplement, chest CT examination may be an important and effective tool for screening, diagnosing, and evaluating the disease severity of COVID-19 in certain circumstances^5,6,8^. There are only a few previous studies describing the temporal changes of COVID-19 on chest CT^4,9–11^.

Huizhou is a city located in the south of China. The confirmed cases of COVID-19 pneumonia were mainly imported from other places with severe epidemic. The assessment of the imaging abnormalities on thin-section CT scans of patients with COVID-19 pneumonia performed during different time periods after illness onset can inform diagnosis and treatment for other locations. In this article, we have examined and provided a description of the natural history of temporal changes in radiological disease on CT scan in patients with COVID-19 pneumonia.

## Results

### Clinical characteristics

The demographic and clinical characteristics of subjects are presented in Table 1. Among the 56 included patients, 26 (46.4%) were males, with a median age of 51 years (range: 1–84 years). We found that 47/56 (83.9%) patients were residents of or had travelled to Hubei Province, while the remaining 9/56 (16.1%) patients reported that they had closely contacted with confirmed or suspected patients with COVID-19. The most prevalent clinical manifestations were fever (66.1%) and cough (53.6%), meanwhile eight patients were asymptomatic on admission. A total of 9/56 (16.1%) patients had at least one underlying disease. The most common abnormalities of laboratory examination were depressed white blood cell (44.6%) and lymphocyte count (42.9%). Only 3/56 (5.3%) included patients were severe cases with COVID-19 pneumonia. At present, all patients have been discharged from hospital, with a median duration of hospitalization of 16.0 (IQR: 12.0–20.0) days.

**Table 1:** Clinical characteristics and main laboratory findings of patients with coronavirus disease 2019 (COVID-19) pneumonia.

Variable	All patients (n = 56)
Age—years	
Median (IQR)	51.0 (37.0–63.0)
Mean ± SD	48.9 ± 15.9
Male sex—no./total no. (%)	26/56 (46.4)
Exposure history within 14 days—no./total no. (%)	
Residents of or having travelled to Hubei Province	47/56 (83.9)
Having closely contacted with confirmed or suspected COVID-19 patients	9/56 (16.1)
Clinical symptoms—no./total no. (%)	48/56 (85.7)
Fever	37/56 (66.1)
Dyspnea	12/56 (21.4)
Cough	30/56 (53.6)
Fatigue	11/56 (19.6)
Myalgia or arthralgia	12/56 (21.4)
Comorbidities—no./total no. (%)	9/56 (16.1)
Hypertension	7/56 (12.5)
Diabetes	3/56 (5.4)
Coronary heart disease	0/56 (0.0)
Chronic obstructive pulmonary disease	1/56 (1.8)
Others	9/56 (16.1)
Clinical classification—no./total no. (%)	
Usual cases	53/56 (94.6)
Severe cases	3/56 (5.4)
Laboratory findings	
White cell count— × 10^9^/L	
Median (IQR)	4.1 (3.2–5.4)
Mean ± SD	4.4 ± 1.8
Distribution –no./total no. (%)	
< 4 × 10^9^/L	3/56 (5.3)
> 10 × 10^9^/L	25/56 (44.6)
Percentage of neutrophils—%	
Median (IQR)	63.4 (55.5–71.0)
Mean ± SD	61.1 ± 15.3
Distribution—no./total no. (%)	
< 40%	6/56 (10.7)
> 70%	16/56 (28.6)
Lymphocyte count—× 10^9^/L	
Median (IQR)	1.1 (0.7–1.4)
Mean ± SD	1.2 ± 0.7
Distribution—no./total no. (%)	
< 0.9 × 10^9^/L	24/56 (42.9)
> 5.2 × 10^9^/L	0/56 (0.0)
Duration of hospitalization—days	
Median (IQR)	16.0 (12.0–20.0)
Mean ± SD	16.6 ± 6.2

Continuous data were summarized as median with interquartile range (IQR) in brackets and mean ± standard deviation (SD). Categorical variables were presented as counts with percentages in brackets.

### Pulmonary CT evaluation

**Figure 1:**
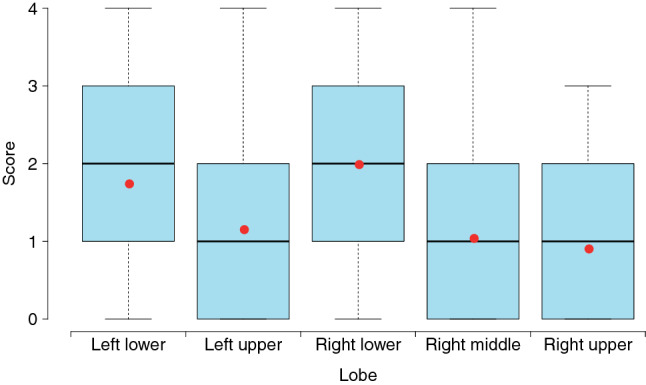
Boxplots of CT scores for different lobes. Thick lines and red points in boxes indicate medians and means of scores, respectively. Lower and upper bounds of boxes represent the 1st (Q1) and 3rd quartiles (Q3) of scores. IQR = Q3-Q1. Thin lines located outside boxes are the minimum and maximum CT scores.

During the course of our study, 141 chest CT scans were performed in the 56 patients. Eleven patients had only one scan, while 21, 13, six and five patients had two, three, four and five scans, respectively. The most common CT abnormalities included ground-glass opacities (GGO) (73.0%), fibrous stripes (39.0%), interlobular septal thickening (31.2%) and consolidation (19.9%), while the atelectasis, pleural or pericardial effusion and thoracic lymphadenopathy were rarely observed (Table 2). As high as 90.8% of the abnormal CT involvement were distributed in bilateral lungs and 124/141 (87.9%) scans suggested more than two lobes involved. Pure peripheral, pure peribronchovascular and diffuse distributions were presented in 67/141 (47.5%), 18/141 (12.8%) and 24/141 (17.0%) CT scans, respectively, while mixed peripheral and peribronchovascular distribution was observed in 32/141 (22.7%) CT scans. CT scores were on average higher in bilateral lower lobes than in other lobes (*P* < 0.001) (Fig. 1).

**Table 2:** Features of thin-section CT scans of patients with coronavirus disease 2019 (COVID-19) pneumonia.

Variable	All scans (n = 141)	Group 1 (n = 23)	Group 2 (n = 42)	Group 3 (n = 45)	Group 4 (n = 31)	*P*
Time from illness onset to CT examination—days						< 0.001
Median (IQR)	15.0 (10.0–21.0)	6.0 (3.0–6.5)	11.0 (10.0–13.0)	18.0 (16.0–20.0)	27.0 (24.0–33.5)	
Mean ± SD	16.3 ± 8.9	4.9 ± 2.0	11.3 ± 2.1	17.9 ± 2.0	29.1 ± 6.7	
Lung lobes involved—no./total no. (%)						< 0.001
Single lobe	17/141 (12.1)	6/23 (26.1)	5/42 (11.9)	2/45 (4.4)	4/31 (12.9)	
Multiple lobes	124/141 (87.9)	17/23 (73.9)	37/42 (88.1)	43/45 (95.6)	27/31 (87.1)	
Lung involvement—no./total no. (%)						0.165
Left	7/141 (5.0)	1/23 (4.3)	2/42 (4.8)	2/45 (4.4)	2/31 (6.5)	
Right	6/141 (4.3)	3/23 (13.0)	3/42 (7.1)	0/45 (0.0)	0/31 (0.0)	
Left and right	128/141 (90.8)	19/23 (82.6)	37/42 (88.1)	43/45 (95.6)	29/31 (93.5)	
Distribution—no./total no. (%)						0.358
Pure peripheral	67/141 (47.5)	13/23 (56.5)	16/42 (38.1)	19/45 (42.2)	19/31 (61.3)	
Pure peribronchovascular	18/141 (12.8)	3/23 (13.0)	8/42 (19.0)	5/45 (11.1)	2/31 (6.5)	
Peripheral and peribronchovascular	32/141 (22.7)	6/23 (26.1)	11/42 (26.2)	11/45 (24.4)	4/31 (12.9)	
Diffuse	24/141 (17.0)	1/23 (4.3)	7/42 (16.7)	10/45 (22.2)	6/31 (19.4)	
Imaging features—no./total no. (%)						
Ground-glass opacities	103/141 (73.0)	19/23 (82.6)	31/42 (73.8)	35/45 (77.8)	18/31 (58.1)	0.035
Halo sign	11/141 (7.8)	5/23 (21.7)	4/42 (9.5)	2/45 (4.4)	0/31 (0.0)	< 0.001
Reversed halo sign	9/141 (6.4)	2/23 (8.7)	3/42 (7.1)	3/45 (6.7)	1/31 (3.2)	–
Interlobular septal thickening	44/141 (31.2)	6/23 (26.1)	15/42 (35.7)	14/45 (31.1)	9/31 (29.0)	0.028
Crazy-paving pattern	21/141 (14.9)	3/23 (13.0)	5/42 (11.9)	7/45 (15.6)	6/31 (19.4)	0.506
Air bronchogram	22/141 (15.6)	5/23 (21.7)	7/42 (16.7)	8/45 (17.8)	2/31 (6.5)	0.163
Consolidation	28/141 (19.9)	5/23 (21.7)	11/42 (26.2)	9/45 (20.0)	3/31 (9.7)	0.190
Subpleural curvilinear line	24/141 (17.0)	3/23 (13.0)	8/42 (19.0)	10/45 (22.2)	3/31 (9.7)	0.004
Fibrous stripes	55/141 (39.0)	5/23 (21.7)	18/42 (42.9)	21/45 (46.7)	11/31 (35.5)	< 0.001
Atelectasis	2/141 (1.4)	1/23 (4.3)	1/42 (2.4)	0/45 (0.0)	0/31 (0.0)	–
Pleural effusion	2/141 (1.4)	1/23 (4.3)	1/42 (2.4)	0/45 (0.0)	0/31 (0.0)	–
Pericardial effusion	4/141 (2.8)	0/23 (0.0)	0/42 (0.0)	2/45 (4.4)	2/31 (6.5)	–
Thoracic lymphadenopathy	3/141 (2.1)	0/23 (0.0)	1/42 (2.4)	0/45 (0.0)	2/31 (6.5)	–

We classified the scans into four groups (Group 1–4) based on the dates on which the CT scans were obtained at the 1st, 2nd, 3rd week or long than three weeks after illness onset. Continuous data were summarized as median with interquartile range (IQR) in brackets and mean ± standard deviation (SD). Categorical variables were presented as counts with percentages in brackets. The linear mixed-effects regression model was applied to compare the time from illness onset to CT examination across four groups. The mixed-effects logistic regression model was used to compare the categorical variables with two levels and with more than 10 scans showing the imaging feature. The Fisher’s exact test was applied to lung involvement and distribution.

**Figure 2:**
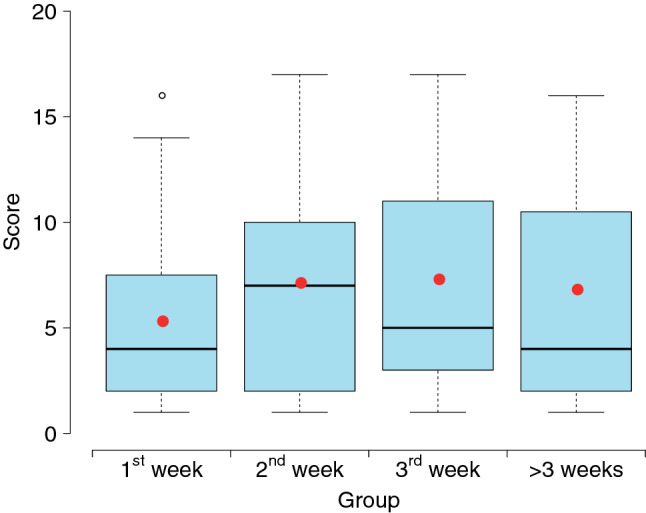
Boxplots of total CT scores for different groups. We classified the CT scans into four groups (Groups 1–4) based on the dates on which the CT scans were obtained at the 1st, 2nd, 3rd week or long than three weeks after illness onset. Thick lines and red points in boxes indicate medians and means of scores, respectively. Lower and upper bounds of boxes represent the 1st (Q1) and 3rd quartiles (Q3) of scores. IQR = Q3-Q1. Thin lines located outside boxes are the minimum total CT scores and the smaller of the maximum total CT scores and Q3 + 1.5 × IQR. The circle indicates the value outside the range between Q1–1.5 × IQR and Q3 + 1.5 × IQR.

**Figure 3:**
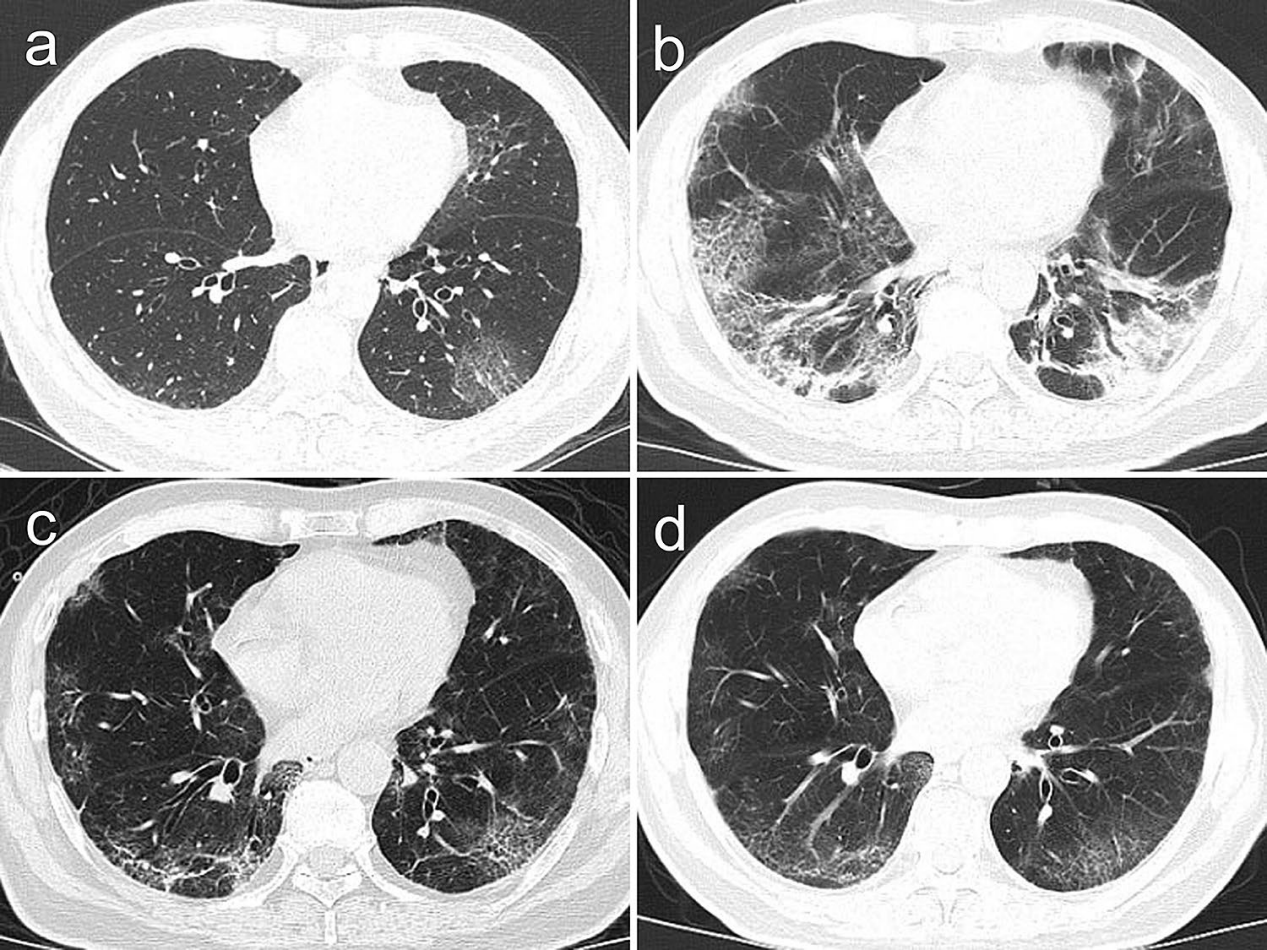
Transverse CT images in a 56-year-old man with COVID-19 pneumonia presented with cough and fever for four days. (**a**) Scans obtained on the 4th day after illness onset showed peripheral patchy ground-glass opacities in the left lower lobe; (**b**) After seven days, the followed-up scan showed lesions progression obviously and consolidation with air bronchogram, ground-glass opacities and crazy paving pattern were observed in the bilateral lungs. (**c**) After 10 days, previous lesions were absorbed obviously and ground-glass opacities, irregular fibrous stripes and interlobular septal thickening were observed in the bilateral lungs. (**d**) After 14 days, further absorption of the lesions was observed. The patient was discharged from hospital three days after the final scan was acquired.

Among the 141 CT scans, 23 (16.3%), 42 (29.8%), 45 (31.9%) and 31 (22.0%) were performed at the 1st, 2nd, 3rd week and longer than three weeks after illness onset (Groups 1–4). A score from 0 to 4 was assigned to each lobe according to the extent of lung involvement of abnormalities. The total CT scores which measured the overall lung involvement were calculated as the sum of CT scores of five lung lobes. It was observed that total CT scores varied across Group 1-Group 4 (*P* < 0.001), with higher median (Group 1: 4; Group 2: 7; Group 3: 5; Group 4: 4) and mean scores (Group 1: 5.3; Group 2: 7.1; Group 3: 7.3; Group 4: 6.8) recorded for scans performed in the 2nd and 3rd week after illness onset (Fig. 2). Imaging features varied across groups whose CT scans were performed at multiple times after illness onset (Table 2 and Fig. 3). GGO predominated in Group 1-Group 4, observed in more than half of the scans, with the lowest percentage occurring in Group 4 (58.1%). The highest percentage of consolidation (26.2%) was observed in scans which were acquired at the 2nd week after illness onset and the percentage declined slightly in Group 4, reaching 9.7%. The percentages for fibrous stripes were statistically significantly different among Group 1-Group 4 (*P* < 0.001), with the highest percentage occurring in Group 3 (46.7%). In Group 4, the percentage of fibrous stripes was as high as 35.5% which followed the proportion of GGO. Most of the CT abnormalities were less common in the scans which were obtained at longer than three weeks after illness onset. Halo sign, reversed halo sign, atelectasis, pleural effusion, pericardial effusion and thoracic lymphadenopathy were relatively rarely presented in the scans (Table 2).

### Follow-up chest CT

In our study, 45 patients had at least two CT scans at different follow-up times, among which 21 (46.7%), 13 (28.9%), 6 (13.3%) and 5 (11.1%) patients had 2–5 scans, with median time intervals between two CT examinations of 6.0, 7.0, 4.5 and 5.8 days, respectively. Among these patients, 11/45 (24.4%), 17/45 (37.8%), 11/45 (24.4%), 6/45 (13.3%) of them were those whose lesions reduced ≥ 75%, 50–75%, 25–50% and < 25% (Group A-Group D), respectively. No patient became worsen. It was observed that the difference in age among Group A-Group D was statistically significant (*P* = 0.005), with the lowest and highest medians of age occurring in Group A (41.0 years) and Group D (64.5 years), respectively. Total CT scores acquired on admission differed across four groups (*P* < 0.001), with the medians increasing from 2.0 for those in Group A to 12.5 for those in Group D. Durations of hospitalization of patients in Group A were on average shorter than in other groups (median duration of hospitalization: Group A: 15.0; Group B: 17.0; Group C: 18.0; Group D: 19.0 days), although the difference was not statistically significant (*P* = 0.595) (Table 3).

**Table 3:** Age, total CT scores on admission and durations of hospitalization of 45 follow-up patients with coronavirus disease 2019 (COVID-19) pneumonia.

Variable	All patients (n = 45)	Group A (n = 11)	Group B (n = 17)	Group C (n = 11)	Group D (n = 6)	*P*
Age—years						0.005
Median (IQR)	51.0 (40.0–63.0)	41.0 (37.0–50.0)	50.0 (37.0–56.0)	63.0 (51.0–65.5)	64.5 (59.5–65.8)	
Mean ± SD	51.2 ± 12.1	43.6 ± 9.6	48.5 ± 11.9	56.9 ± 11.3	61.8 ± 6.2	
Total CT score on admission—points						< 0.001
Median (IQR)	6.0 (3.0–10.0)	2.0 (1.0–4.0)	4.0 (3.0–7.0)	9.0 (7.5–13.0)	12.5 (7.8–15.8)	
Mean ± SD	6.7 ± 4.9	2.9 ± 2.3	5.4 ± 3.6	10.1 ± 4.5	11.2 ± 5.4	
Duration of hospitalization—days						0.595
Median (IQR)	17.0 (14.0–20.0)	15.0 (13.0–18.5)	17.0 (14.0–20.0)	18.0 (16.5–19.0)	19.0 (15.8–23.8)	
Mean ± SD	17.8 ± 6.1	15.7 ± 3.6	18.2 ± 7.2	17.5 ± 4.0	21.0 ± 9.1	

We categorized the 45 patients who had at least two follow-up scans into four groups: Group A, patients whose lesions reduced ≥ 75%; Group B, those whose lesions reduced 50–75%; Group C, those whose lesions reduced 25–50%; Group D, those whose lesions reduced < 25%. Continuous data were summarized as median with interquartile range (IQR) in brackets and mean ± standard deviation (SD). The Kruskal–Wallis test was applied to compare continuous variables across four groups.

Results of sensitivity analysis were consistent with those of the main analysis regarding the differences in the imaging features of CT scans acquired at the 1st, 2nd, 3rd week and longer than three weeks after illness onset, suggesting that the findings were robust to the exclusion of the eight patients who did not present with symptoms on admission (Supplementary Material Fig. S1 and Table S1).

## Discussion

The present study examined imaging abnormalities on thin-section CT scans performed during different time periods after illness onset for patients with COVID-19 pneumonia reported in Huizhou, China. We found that GGO, bilateral multiple lobes, peripheral with or without peribronchovascular distribution, with preference in lower lobes were the most common CT findings in COVID-19 pneumonia, which were similar to those reported previously for patients with COVID-19 pneumonia, SARS and MERS^10,12–14^. It was not surprising to observe the similar imaging features in patients with COVID-19 pneumonia, SARS and MERS, since all of them are family members of coronaviruses.

In our study, it was found that the most common clinical manifestations of patients with COVID-19 pneumonia were fever and cough, which was consistent with the findings reported previously for COVID-19 pneumonia^15^ and other lower respiratory tract infections^10^. It was worth noting that eight patients were asymptomatic at the early stage of SARS-CoV-2 infections but had abnormal CT manifestations, indicating that clinical symptoms may be inconsistent with radiological findings to some extent^16^. In addition, nucleic acid test has a high false negative rate. Therefore, performing CT scans might be beneficial for screening patients with COVID-19 at the early stage if the CT examination is feasible. However, imaging is not always recommended. The usage of CT examination should account for the severity of a patient, the availability of COVID-19 test result, health-care facility and personnel^8^.

GGO was the most predominant pattern presented in chest CT scans of patients with COVID-19 pneumonia. It was a common abnormality for viral pneumonias, but not specific to COVID-19^17^. GGO could be caused by various of factors, such as partial collapse of alveoli, interstitial thickening (due to fluid, cells, and/or fibrosis), partial filling of airspaces, and increased capillary blood volume, or a combination of these, with the common factor being the partial displacement of air^18^. A SARS-CoV-2 infection can be followed by a severe lower respiratory tract infection with GGO or consolidation or both, which is similar to the organizing pneumonia^19^. Severe acute respiratory syndrome coronavirus (SARS-CoV), Middle East respiratory syndrome coronavirus (MERS-CoV) and influenza infections can lead to secondary organizing pneumonia^11^. Peripheral distribution of lung abnormalities was the dominant distribution pattern of patients with COVID-19 reported in most of the previous studies^10,16^. However, distribution along bronchovascular bundle was also common in the CT scans of our subjects, which suggested that lung abnormalities may spread from periphery to central region. Meanwhile, we cannot rule out the possibility that central abnormalities occur in COVID-19.

We observed that the extent of lung abnormalities in patients with COVID-19 pneumonia at the 2nd and 3rd week after illness onset were the most serious than those performed at other time points. The findings were partially in accordance with three earlier studies^4,10,20^. We found that the main abnormalities presented on CT scans were GGO, interlobular septal thickening and fibrous stripes during the 1st week, which suggested the lung parenchymal and interstitial injury at the relatively early stage. The total CT scores in the 2nd and 3rd week were higher compared with those recorded in the 1st and 4th week. Meanwhile, the frequency of consolidation was also elevated in the 2nd and 3rd week, although the differences in the frequency across Groups 1–4 were not statistically significant. These findings consistently suggested the disease progression of COVID-19 in the 2nd and 3rd week. Total CT scores on average reduced in the patients whose CT scans were obtained longer than three weeks after illness onset, meanwhile GGO and consolidation declined in frequency apparently, implying the decreasing extent of lung abnormalities in this time period. Interestingly, the frequency of fibrous stripes was relatively higher in this time period compared with other imaging features, following the frequency of GGO. Fibrous stripes may occur during the healing of lung inflammation or proliferative diseases, with scar tissues replacing cellular components gradually^21^. The presence of fibrous stripes sometimes indicates the improvement in prognosis of a COVID-19 patient with stabilizing disease status^5^.

Serial CT scans in our study provided a precious opportunity to monitor lung changes in COVID-19 patients. We found that the median hospitalization duration was shorter in patients whose lesions reduced ≥ 75% than others, although the difference in the hospitalization duration was not statistically significant. Relatively small sample size may account for the non-significant disparity. In addition, our findings suggested that the age and extent of lung abnormalities on admission could affect the evolution of lesions in lung. In our study, we found that most of the 45 follow-up patients recovered with residual lesions, suggesting patients meeting the criteria to discharge does not mean that they recovered completely. In addition, it has been reported that some patients discharged from hospital were testing positive for SARS-CoV-2 several days after being discharged^22^. And thus, we recommend review for COVID-19 patients who have been discharged from hospital and the patients may need isolation. Despite the 100% discharge rate, a wide spectrum of radiological disease burden was observed, suggesting that this may be poorly predictive of prognosis.

There were several limitations in our study. First, we had a relatively small number of patients included in this study due to the limited number of patients identified in Huizhou. Second, we have only three severe cases in our cohort. Third, 46.7% of the included patients with COVID-19 pneumonia had only two CT scans. Although most of the patients have been discharged from hospital after two CT scans were acquired, more CT scans could help for evaluating whether the residual lesions were absorbed in the future. Fourth, as this was a retrospective cohort study, there was inherent selection bias of patients, both in terms of who received a CT scan and how many scans were performed. Next, the relationship between imaging and histopathological findings was not assessed, since lung biopsy specimens were not available in our study. Further, the time intervals between two CT scan acquisitions varied across patients since we could not require all of the patients to have systematically evaluations at certain times. Finally, we are unable to confirm whether the radiological changes described resolve completely as patients have not been followed up at a later date after discharge. Further studies can be conducted to examine the radiological changes of COVID-19 patients after discharge.

In conclusion, the predominant imaging features of COVID-19 pneumonia are GGO, bilateral multiple lobular, peripheral with or without peribronchovascular distribution, with preference in lower lobes. There are temporal patterns of lung abnormalities, with higher extent of involvement in the 2nd and 3rd week. Review are recommended for COVID-19 patients who have been discharged from hospital and the patients may need isolation.

## Methods

The current study was reviewed and approved by the Research Ethics Committee of the Central People’s Hospital of Huizhou. All methods were carried out in accordance with guidelines outlined in the Declaration of Helsinki. The requirement for individual patient approval for participation deemed not to be required. Informed consent was waived by the ethic committee. We collected and analyzed data of patients anonymously.

### Patients

All of the patients confirmed with COVID-19, including symptomatic and asymptomatic cases, were required to be isolated in China during the study period^23^ and the Central People’s Hospital of Huizhou was the place for isolation in Huizhou. Patients with COVID-19 pneumonia in Huizhou city between January 22, 2020 and March 5, 2020 were under treatment in the Central People’s Hospital of Huizhou. These patients were diagnosed with COVID-19 according to the World Health Organization (WHO) interim guidance^24^. A total of 62 consecutive patients in the hospital were enrolled in this retrospective study. Among the 62 patients, three patients did not have chest CT scans, while another three patients did not have abnormal chest CT imaging. We excluded the six patients and the remaining 56 patients were included. Results of SARS-CoV-2 tests were negative for three patients on admission and positive results were obtained after a few days. All patients enrolled have been discharged from hospital according to the discharge criteria formulated by National Health Commission^25,26^.

Data on sex, age, clinical symptoms, laboratory findings, exposure history (resident of or having travelled to Hubei Province; having closely contacted with confirmed or suspected COVID-19 patients), comorbidities, the date of illness onset and the date of performing CT examination were collected for all patients by carefully reviewing their medical records. Severe cases were those with any of the following features: (1) serious complications (e.g. respiratory failure, other organ failure, septic shock), (2) respiratory rate ≥ 30 breaths per minute, (3) oxygen saturation at rest ≤ 93%, (4) the ratio of the partial pressure of arterial oxygen to the fraction of inspired oxygen ≤ 300 mmHg^26^. Disease duration was defined as the time interval between the date of symptom onset or on which the specimen of asymptomatic case was detecting positive for SARS-CoV-2 and the date of CT acquisition.

### CT Protocol

Chest CT scans were obtained on a commercial 16-detector CT scanner (Philips MX16, Philips Medical Systems, the Netherlands). To reduce motion artifacts, the patients were required to hold breath during scanning. All of the patients were performed a spiral scan for the following parameters: 2-mm reconstructed slice thickness, 1-mm interval, 24-mmcollimation, 360-mm field of view, 512-mm matrix, 120 kV, automatic tube current modulation. From the raw data, CT images were reconstructed with a lung algorithm for parenchymal analysis. The reconstructed images were then sent to the workstation and picture archiving and communication systems (PACS) for multiplanar reconstruction.

### Imaging analysis

All images were reviewed by two experienced chest radiologists who have worked for more than five years in this field blindly to the clinical information. They reviewed the images independently and reached a decision in consensus. The following image characteristics were recorded^18^: (1) GGO (defined as hazy increased opacity of lung without obscuration of the underlying vessels and airway walls), (2) consolidation (defined as homogeneous increase of lung parenchyma with obscuration of the underlying vessels and airway walls), (3) crazy-paving pattern (defined as GGO with superimposed thickened interlobular and intralobular lines), (4) interlobular septal thickening, (5) halo sign (defined as a nodule or mass surrounded by GGO), (6) reversed halo sign (defined as focal rounded area of GGO surrounded by a more or less complete ring of consolidation), (7) fibrous stripes (linear opacities of lung, as shown in Supplementary Material Fig. S2), (8) air bronchogram, (9) atelectasis, (10) subpleural curvilinear line, (11) pleural or pericardial effusion, (12) thoracic lymphadenopathy (defined as lymphnode with short axis diameter ≥ 1 cm). To estimate the extent of lung involvement of all of these abnormalities, we used a semi-quantitative score to assign each of five lung lobes. Each lobe was assigned a score from 0 to 4 (0: no involvement; 1: 1–25% involvement; 2: 26–49% involvement; 3: 50–75% involvement; 4: > 75% involvement)^9,12^. The CT scores of five lung lobes were summed up as the total CT score which measured the overall lung involvement, ranging from 0 (no involvement) to 20 (maximum involvement).

**Figure 4:**
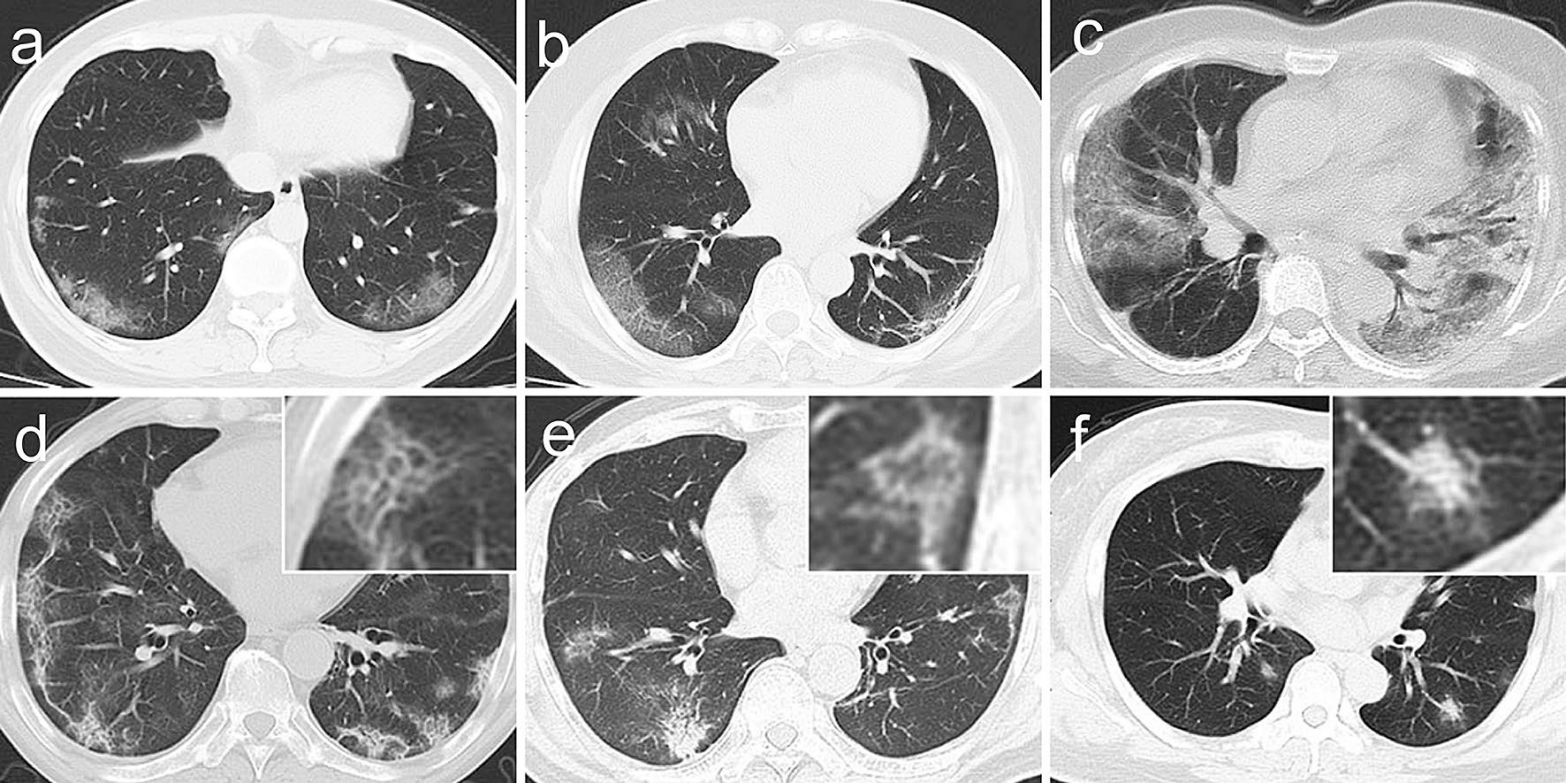
Chest CT findings of COVID-19 pneumonia on transaxial images. (**a**) Bilateral and peripheral ground-glass opacities in the lower lobe; (b) Ground-glass opacities along peripheral and peribronchovascular distribution in the right lower and middle lobes, subpleural curvilinear line in the left lower lobes; (**c**) Consolidation with air bronchogram in the left lung; (**d**) Crazy paving pattern along peripheral distribution in bilateral lungs; (**e**) Reversed halo sign in the left lower lobe; (**f**) Halo sign in the left lower lobe.

Distributions of lung abnormalities were divided into four types: (1) peripheral (involving mainly the outer one-third of the lung), (2) peribronchovascular (abnormalities along the path way of bronchovascular bundle), (3) diffuse (continuous involvement without respect to lung segments), (4) both peripheral and peribronchovascular. Radiological manifestations and distribution of some glossaries were presented in Fig. 4.

Subsequently, we classified the CT scans of 56 patients into four groups based on the dates on which the CT scans were obtained at the 1st, 2nd, 3rd week or longer than three weeks after illness onset. If a patient was asymptomatic, we categorized the scan of the patient according to the date on which the specimen of patient was detecting positive for SARS-CoV-2 instead of the date of symptom onset.

Based on the extent of reduction in abnormities presenting in CT imagines, we categorized the 45 patients which had at least two follow-up CT scans into four groups: Group A, patients whose lesions reduced ≥ 75%; Group B, those whose lesions reduced 50–75%; Group C, those whose lesions reduced 25–50%; Group D, those whose lesions reduced < 25%).

### Statistical analysis

We summarized continuous variables as medians and interquartile ranges (IQRs) or ranges as appropriate and mean ± standard deviation (SD), while categorical variables were expressed as counts and percentages. We used mixed-effects models to compare the features of 141 scans of 56 patients for different lobes and across Groups 1–4, accounting for the clustering effect of patient. Specifically, cumulative link mixed models were fitted for the comparisons of CT scores of different lobes and total CT scores of Groups 1–4. The linear mixed-effects regression model was applied to the time from illness onset to CT examination. Mixed-effects logistic regression models were used to compare the categorical variables with two levels and with more than 10 scans showing the imaging feature across Group 1-Group 4. The Fisher’s exact test was applied to lung involvement and distribution, since mixed-effects models cannot be fitted with the data of these variables. We used the Kruskal–Wallis test to compare age, total CT scores on admission and durations of hospitalization across Groups A–D, since the number of data points was relatively small, and it seemed that the values of these variables were not normally distributed (Supplementary Material Fig. S3).

Sensitivity analysis was conducted to compare the imaging features of CT scans across Group 1-Group 4 for the 48 patients who presented with symptoms on admission, avoiding the potential influence of different definitions of the duration from illness onset to CT examination between patients that showed symptoms on admission and those who did not. All statistical analyses were performed using R software version 3.6.2 (R Foundation for Statistical Computing).

## Supplementary information

Supplementary information.

## Data Availability

The datasets generated during and/or analysed during the current study are not publicly available due to the data sharing policy of the Central People’s Hospital of Huizhou, but are available from the corresponding author on reasonable request.

## References

[1] World Health Organization. Coronavirus disease 2019 (COVID-19) Situation Report-159. https://www.who.int/docs/default-source/coronaviruse/situation-reports/20200627-covid-19-sitrep-159.pdf?sfvrsn=93e027f6_2 (2020).

[2] Chung M (2020). CT imaging features of 2019 novel Coronavirus (2019-nCoV). Radiology.

[3] Huang C (2020). Clinical features of patients infected with 2019 novel coronavirus in Wuhan China. Lancet.

[4] Pan F (2020). Time course of lung changes on chest CT during recovery from 2019 novel Coronavirus (COVID-19) pneumonia. Radiology.

[5] Pan Y (2020). Initial CT findings and temporal changes in patients with the novel coronavirus pneumonia (2019-nCoV): a study of 63 patients in Wuhan China. Eur Radiol.

[6] Diao K, Han P, Pang T, Li Y, Yang Z (2020). HRCT imaging features in representative imported cases of 2019 novel coronavirus pneumonia. Precis. Clin. Med..

[7] Xie X (2020). Chest CT for typical 2019-nCoV pneumonia: relationship to negative RT-PCR testing. Radiology.

[8] Rubin GD (2020). The role of chest imaging in patient management during the COVID-19 pandemic: a multinational consensus statement from the Fleischner society. Radiology.

[9] Liang T (2020). Evolution of CT findings in patients with mild COVID-19 pneumonia. Eur. Radiol..

[10] Shi H (2020). Radiological findings from 81 patients with COVID-19 pneumonia in Wuhan, China: a descriptive study. Lancet Infect. Dis..

[11] Wang Y (2020). Temporal changes of CT findings in 90 patients with COVID-19 pneumonia: a longitudinal study. Radiology.

[12] Ooi GC (2004). Severe acute respiratory syndrome: temporal lung changes at thin-section CT in 30 patients. Radiology.

[13] Ajlan AM, Ahyad RA, Jamjoom LG, Alharthy A, Madani TA (2014). Middle east respiratory syndrome coronavirus (MERS-CoV) infection: chest CT findings. AJR Am. J. Roentgenol..

[14] Wong KT (2003). Thin-section CT of severe acute respiratory syndrome: evaluation of 73 patients exposed to or with the disease. Radiology.

[15] Chen N (2020). Epidemiological and clinical characteristics of 99 cases of 2019 novel coronavirus pneumonia in Wuhan, China: a descriptive study. Lancet.

[16] Xu X (2020). Imaging and clinical features of patients with 2019 novel coronavirus SARS-CoV-2. Eur. J. Nucl. Med. Mol. Imaging.

[17] Kim EA (2002). Viral pneumonias in adults: radiologic and pathologic findings. Radiographics.

[18] Hansell DM (2008). Fleischner society: glossary of terms for thoracic imaging. Radiology.

[19] Kanne JP, Little BP, Chung JH, Elicker BM, Ketai LH (2020). Essentials for radiologists on COVID-19: an update-radiology scientific expert panel. Radiology.

[20] Bernheim A (2020). Chest CT findings in coronavirus disease-19 (COVID-19): relationship to duration of infection. Radiology.

[21] Ye Z, Zhang Y, Wang Y, Huang Z, Song B (2020). Chest CT manifestations of new coronavirus disease 2019 (COVID-19): a pictorial review. Eur. Radiol..

[22] Zhang JF (2020). SARS-CoV-2 turned positive in a discharged patient with COVID-19 arouses concern regarding the present standard for discharge. Int. J. Infect. Dis..

[23] Disease Prevention and Control Bureau, National Health Commission of the People’s Republic of China. Management of asymptomatic cases of COVID-19. https://www.nhc.gov.cn/jkj/s7916/202004/9d3edaaebb9a4c369f42c61039be35fe.shtml (2020). 10.46234/ccdcw2020.082 PMC8392946 34594648

[24] World Health Organization. Clinical management of severe acute respiratory infection when novel coronavirus (2019-nCoV) infection is suspected. https://www.who.int/docs/default-source/coronaviruse/clinical-management-of-novel-cov.pdf?sfvrsn=bc7da517_2 (2020).

[25] National Health Commission of the People’s Republic of China. Diagnosis and treatment protocols of pneumonia caused by a novel coronavirus (trial version 5). https://117.128.6.26/cache/www.nhc.gov.cn/yzygj/s7653p/202002/3b09b894ac9b4204a79db5b8912d4440/files/7260301a393845fc87fcf6dd52965ecb.pdf?ich_args2=465-18204219015589_8c563b9f890ddd26f265c52c792d732e_10001002_9c896c28d2c0f4d29133518939a83798_88ccd8b555f083c7147cc824123d01d8 (2020).

[26] National Health Commission of the People’s Republic of China. Diagnosis and treatment protocols of pneumonia caused by a novel coronavirus (trial version 6). https://www.nhc.gov.cn/yzygj/s7653p/202002/8334a8326dd94d329df351d7da8aefc2/files/b218cfeb1bc54639af227f922bf6b817.pdf (2020).

