# Publisher Correction: Temporal lung changes on thin-section CT in patients with COVID-19 pneumonia

**DOI:** 10.1038/s41598-021-84454-9

**Published:** 2021-02-23

**Authors:** Zhiyan Zhang, Runhui Tang, Heyang Sun, Haiyang Dai, Kangyin Chen, Xinmiao Ye, Wei Ye, Shengkai Li, Bowen Lan, Li Li, Chun-Quan Ou

**Affiliations:** 1 Department of Medical Imaging, The Central People’s Hospital of Huizhou, No. 41, Eling Road North, Huicheng District, Huizhou, 516001 Guangdong Province China; 2 Department of Urology, The Central People’s Hospital of Huizhou, No. 41, Eling Road North, Huicheng District, Huizhou, Guangdong Province China; 3 State Key Laboratory of Organ Failure Research, Department of Biostatistics, Guangdong Provincial Key Laboratory of Tropical Disease Research, School of Public Health, Southern Medical University, No. 1023, Shatai Road South, Baiyun District, Guangzhou, 510515 Guangdong Province China

Correction to: *Scientific Reports* 10.1038/s41598-020-76776-x, published online 12 November 2020

The original version of this Article contained a typographical error in the jointly supervising statement.

“The author jointly supervised this work: Bowen Lan, Li Li, and Chun-Quan Ou.”

now reads:

“These authors jointly supervised this work: Bowen Lan, Li Li, and Chun-Quan Ou.”

This error has now been corrected in the PDF and HTML versions of the Article.

