# Supplementary material for: Temporal lung changes on thin-section CT in patients with COVID-19 pneumonia
Source: Sci Rep. 2020 Nov 12;10:19649. doi: 10.1038/s41598-020-76776-x (PMC7661534; doi:10.1038/s41598-020-76776-x)
Supplement: Supplementary file 1 — Supplementary information. [file 41598_2020_76776_MOESM1_ESM.docx]

# Supplementary Material

# Temporal lung changes on thin-section CT in patients with COVID-19 pneumonia

Zhiyan Zhang^1#^, Runhui Tang^1#^, Heyang Sun^2^, Haiyang Dai^1^, Kangyin Chen^1^, Xinmiao Ye^1^, Wei Ye^1^, Shengkai Li^1^, Bowen Lan^1*^, Li Li^3*^, Chun-Quan Ou^3†^

^1^ Department of Medical Imaging, The Central People’s Hospital of Huizhou, No. 41, Eling Road North, Huicheng District, Huizhou, 516001, Guangdong Province, China

^2^ Department of Urinary Surgery, The Central People’s Hospital of Huizhou, No. 41, Eling Road North, Huicheng District, Huizhou, Guangdong Province, China

^3^ State Key Laboratory of Organ Failure Research, Department of Biostatistics, Guangdong Provincial Key Laboratory of Tropical Disease Research, School of Public Health, Southern Medical University, No. 1023, Shatai Road South, Baiyun District, Guangzhou, 510515, Guangdong Province, China

^#^ Joint first authors

* Joint corresponding authors:

Li Li, email: [lylygdsg@163.com](mailto:lylygdsg@163.com)

Bowen Lan, email: [lanbowenbw@163.com](mailto:lanbowenbw@163.com)

^†^ Senior author

**Supplementary Material Figure S1.** Boxplots of total CT scores across different groups used in the sensitivity analysis. We classified total CT scans into four groups (Groups 1-4) based on the dates on which the CT scans were obtained at the 1^st^, 2^nd^, 3^rd^ week or long than three weeks after illness onset. Thick lines and red points in boxes indicate medians and means of scores, respectively. Lower and upper bounds of boxes represent the 1^st^ (Q1) and 3^rd^ quartiles (Q3) of total CT scores. IQR=Q3-Q1. Thin lines located outside boxes are the minimum total CT scores and the smaller of the maximum total CT scores and Q3+1.5🞨IQR. Circles were used to indicate values outside the ranges between Q1-1.5🞨IQR and Q3+1.5🞨IQR.

**
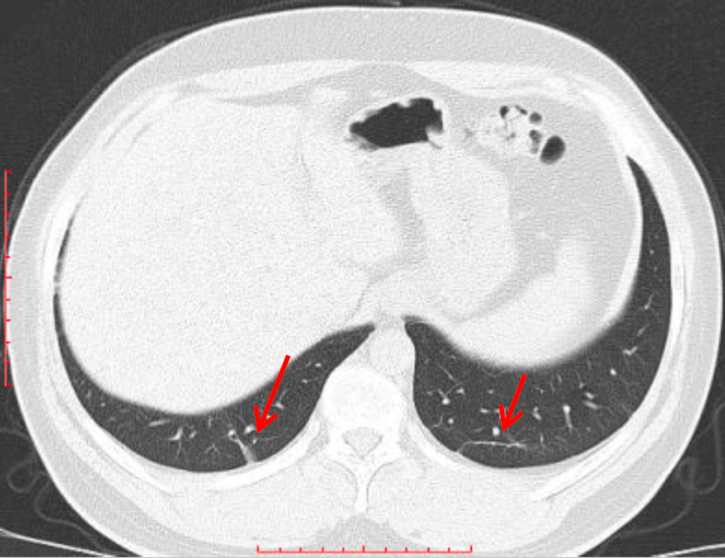
**

**Supplementary Material Figure S2**. Image of a CT scan showing fibrous stripes.

**Supplementary Material Figure S3.** Histograms of age and total CT scores on admission and durations of hospitalization of the 45 follow-up patients.

**Supplementary Material Table S1.** Features of thin-section CT scans of patients with coronavirus disease 2019 (COVID-19) pneumonia in the sensitivity analysis.

| Variable | All scans (n=127) | Group 1 (n=19) | Group 2 (n=35) | Group 3 (n=42) | Group 4 (n=31) | *P* |
| --- | --- | --- | --- | --- | --- | --- |
| Time from illness onset to CT  examination – days |  |  |  |  |  | <0.001 |
| Median (IQR) | 16.0 (10.5–21.0) | 6.0 (3.5– 7.0) | 11.0 (10.0–13.5) | 18.0 (16.0–20.0) | 27.0 (24.0–33.5) |  |
| Mean ± SD | 16.9 ± 8.9 | 5.1 ± 2.1 | 11.4 ± 2.1 | 17.9 ± 2.1 | 29.1 ± 6.7 |  |
| Lung lobes involved – no./total no. (%) |  |  |  |  |  | <0.001 |
| Single lobe | 13/127 (10.2) | 4/19 (21.1) | 3/35 (8.6) | 2/42 (4.8) | 4/31 (12.9) |  |
| Multiple lobes | 114/127 (89.8) | 15/19 (78.9) | 32/35 (91.4) | 40/42 (95.2) | 27/31 (87.1) |  |
| Lung involvement – no./total no. (%) |  |  |  |  |  | 0.057 |
| Left | 5/127 (3.9) | 0/19 (0.0) | 1/35 (2.9) | 2/42 (4.8) | 2/31 (6.5) |  |
| Right | 6/127 (4.7) | 3/19 (15.8) | 3/35 (8.6) | 0/42 (0.0) | 0/31 (0.0) |  |
| Left & right | 116/127 (91.3) | 16/19 (84.2) | 31/35 (88.6) | 40/42 (95.2) | 29/31 (93.5) |  |
| Distribution – no./total no. (%) |  |  |  |  |  | 0.349 |
| Pure peripheral | 59/127 (46.5) | 11/19 (57.9) | 12/35 (34.3) | 17/42 (40.5) | 19/31 (61.3) |  |
| Pure peribronchovascular | 16/127 (12.6) | 2/19 (10.5) | 7/35 (20.0) | 5/42 (11.9) | 2/31 (6.5) |  |
| Peripheral & peribronchovascular | 28/127 (22.0) | 5/19 (26.3) | 9/35 (25.7) | 10/42 (23.8) | 4/31 (12.9) |  |
| Diffuse | 24/127 (18.9) | 1/19 (5.3) | 7/35 (20.0) | 10/42 (23.8) | 6/31 (19.4) |  |
| Imaging features – no./total no. (%) |  |  |  |  |  |  |
| Ground-glass opacities | 93/127 (73.2) | 16/19 (84.2) | 27/35 (77.1) | 32/42 (76.2) | 18/31 (58.1) | 0.021 |
| Halo sign | 10/127 (7.9) | 4/19 (21.1) | 4/35 (11.4) | 2/42 (4.8) | 0/31 (0.0) | - |
| Reversed halo sign | 9/127 (7.1) | 2/19 (10.5) | 3/35 (8.6) | 3/42 (7.1) | 1/31 (3.2) | - |
| Interlobular septal thickening | 42/127 (33.1) | 6/19 (31.6) | 14/35 (40.0) | 13/42 (31.0) | 9/31 (29.0) | 0.017 |
| Crazy-paving pattern | 20/127 (15.7) | 3/19 (15.8) | 5/35 (14.3) | 6/42 (14.3) | 6/31 (19.4) | 0.690 |
| Air bronchogram | 20/127 (15.7) | 5/19 (26.3) | 6/35 (17.1) | 7/42 (16.7) | 2/31 (6.5) | 0.143 |
| Consolidation | 26/127 (20.5) | 4/19 (21.1) | 10/35 (28.6) | 9/42 (21.4) | 3/31 (9.7) | 0.147 |
| Subpleural curvilinear line | 23/127 (18.1) | 3/19 (15.8) | 8/35 (22.9) | 9/42 (21.4) | 3/31 (9.7) | 0.003 |
| Fibrous stripes | 52/127 (40.9) | 4/19 (21.1) | 17/35 (48.6) | 20/42 (47.6) | 11/31 (35.5) | 0.001 |
| Atelectasis | 2/127 (1.6) | 1/19 (5.3) | 1/35 (2.9) | 0/42 (0.0) | 0/31 (0.0) | - |
| Pleural effusion | 2/127 (1.6) | 1/19 (5.3) | 1/35 (2.9) | 0/42 (0.0) | 0/31 (0.0) | - |
| Pericardial effusion | 4/127 (3.1) | 0/19 (0.0) | 0/35 (0.0) | 2/42 (4.8) | 2/31 (6.5) | - |
| Thoracic lymphadenopathy | 3/127 (2.4) | 0/19 (0.0) | 1/35 (2.9) | 0/42 (0.0) | 2/31 (6.5) | - |

Notes: We classified the scans into four groups (Group 1-4) based on the dates on which the CT scans were obtained at the 1^st^, 2^nd^, 3^rd^ week and long than three weeks after illness onset. Continuous data were summarized as median with interquartile range (IQR) in brackets and mean ± standard deviation (SD). Categorical variables were presented as counts with percentages in brackets. The linear mixed-effects regression model was applied to compare the time from illness onset to CT examination among four groups. The mixed-effects logistic regression model was used to compare the categorical variables with two levels and with more than 10 scans showing the imaging feature. The Fisher’s exact test was applied to lung involvement and distribution.
